# Construction of a comprehensive value assessment framework for renal denervation therapy: a decision-support tool for Chinese hospital management

**DOI:** 10.3389/fpubh.2026.1801333

**Published:** 2026-06-05

**Authors:** Chang Liu, Min Lin, Feng Xu, Yi Yan, Libo Tao

**Affiliations:** 1 Center for Health Policy and Technology Evaluation, Peking University Health Science Center, Beijing, China; 2 The Second Affiliated Hospital, Zhejiang University School of Medicine, Hangzhou, China; 3 Peking University Third Hospital, Beijing, China; 4 Department of Health Policy and Management, Peking University Health Science Center, Beijing, China

**Keywords:** comprehensive value assessment framework, health technology assessment, hospital management, MCDA, RDN

## Abstract

**Objective:**

Renal denervation (RDN) therapy is an innovative interventional therapy for patients with refractory hypertension and antihypertensive drug intolerance. In China, four RDN products were approved by National Medical Products Administration. This study aims to construct a comprehensive value assessment framework for RDN therapy to support the rational selection and application in Chinese hospitals.

**Methods:**

Multiple Criteria Decision Analysis (MCDA) approach was used to develop the framework. We conducted a literature review and focus group interviews to identify the list of criteria that should be included in the framework. Then, the Delphi method was applied to refine the framework. With the engagement of cardiologists and non-clinical professionals from hospitals in different regions, weights were given to each criterion by the Analytic Hierarchy Process.

**Results:**

The comprehensive value assessment framework for RDN therapy consists of three levels of criteria in seven domains, including seven criteria at the first level, 16 secondary criteria and 30 sub-criteria at the third level. The distribution of criteria weights at the first level is: efficacy (23.26%), safety (21.83%), qualification and suitability (16.84%), patient value (15.18%), innovation (10.65%), management value (6.22%), and cost (6.01%). The prioritized domains are efficacy, safety, qualification and suitability.

**Conclusion:**

The development of a comprehensive value assessment framework for RDN can serve as a reliable and scientific tool to support the procurement reviews and decisions for RDN devices in Chinese hospitals.

## Introduction

Drug value assessment has evolved into a multidisciplinary field. In practice, it draws on methodologies including Health Technology Assessment (HTA), pharmacoeconomic evaluation, and multi-criteria decision analysis (MCDA) (1). According to the international HTA definition, it covers not only clinical efficacy but also economic, ethical, social, and organizational dimensions to support transparent healthcare decisions (2). In China, a preliminary framework for drug value assessment has been established, including a two-stage HTA process for national reimbursement decisions (3) and the 2021 Guidelines for Comprehensive Clinical Evaluation of Drugs, which promote multi-dimensional assessment for rational use and supply sustainability (4). This mechanism incorporates clinical, economic and societal factors, which not only supports healthcare and reimbursement decisions but also improves transparency and equity in health care decision-making, a development that has been highly recognized by Chinese policymakers. Nevertheless, while drug value assessment has made significant strides, the assessment of medical device value in China significantly lags behind. This disparity is largely attributable to the distinctive characteristics of medical devices, which include shorter product lifecycles, challenges in generating classical clinical trial data, and dynamic interactions between devices and operators (5).

Recently, there has been a growing demand to develop the value assessment mechanism for medical devices, especially among hospital procurement decision-makers. A large number of medical devices were subject to incremental updates and marketed as “innovations.” The evaluation of medical device innovations presented challenges for hospital medical device management. In 2025, over 40 Chinese medical devices gained breakthrough designations from Food and Drug Administration (FDA). Over 500 imported and local medical devices obtained innovation designations from China National Medical Products Administration (NMPA) annually. Also, with the requirements for further medical cost reductions and health budget constraints, a widespread concern is that public hospitals become less incentivized to adopt costly but innovative technologies. Under this circumstance, to balance clinical development, patient needs and hospital resource allocation, the comprehensive value assessment approach for medical devices is greatly needed to guide product selection in hospital settings.

Multiple Criteria Decision Analysis (MCDA) has been increasingly adopted as an evidence-based decision support tool in the context of healthcare management (6–9). It enables structured decision-making through transforming complex evaluations into well-defined criteria, followed by systematic weighting and scoring based on their predetermined importance hierarchy (8). This makes MCDA particularly well-suited to tackling the challenges outlined in the subsequent section, such as reconciling the diverse focuses of various stakeholders and integrating multi-dimensional value aspects beyond clinical efficacy alone. Therefore, this study utilizes renal denervation (RDN) as an illustrative case to construct a tailored comprehensive value assessment framework by using MCDA to evaluate innovative medical devices. The aim is to support more transparent, accountable and evidence-informed product selection and rational use in clinical settings.

RDN is an innovative catheter-based intervention that delivers targeted ablation energy to the renal sympathetic nerves, disrupting the overactive sympathetic signaling between the kidneys and the central nervous system to reduce blood pressure (10–12). In 2024, four RDN devices were approved by NMPA successively, including one imported product and three domestic products. Because RDN therapy has been commercially available in China for about a year, physicians and hospital administrators have limited experience with clinical use and operational management. Besides, these devices differ in design features, ease of use and the richness of clinical evidence. Manufacturers also vary in their training programs and technical support capabilities, which may contribute to heterogeneity in procedural performance and outcomes across hospitals. Consequently, physicians and hospital administrators often find it difficult to compare and select RDN devices based on a single dimension, such as cost-effectiveness or clinical efficacy. As both clinical and non-clinical stakeholders are involved in the hospital procurement process and may prioritize different value aspects, the absence of a structured and consistent evaluation approach can make decision-making time-consuming, less transparent, and difficult to justify.

To address these challenges, this study applies the MCDA approach to establish a comprehensive value assessment framework for RDN, aiming to support more transparent and evidence-informed hospital procurement and management decisions.

## Methods

**Figure 1:**
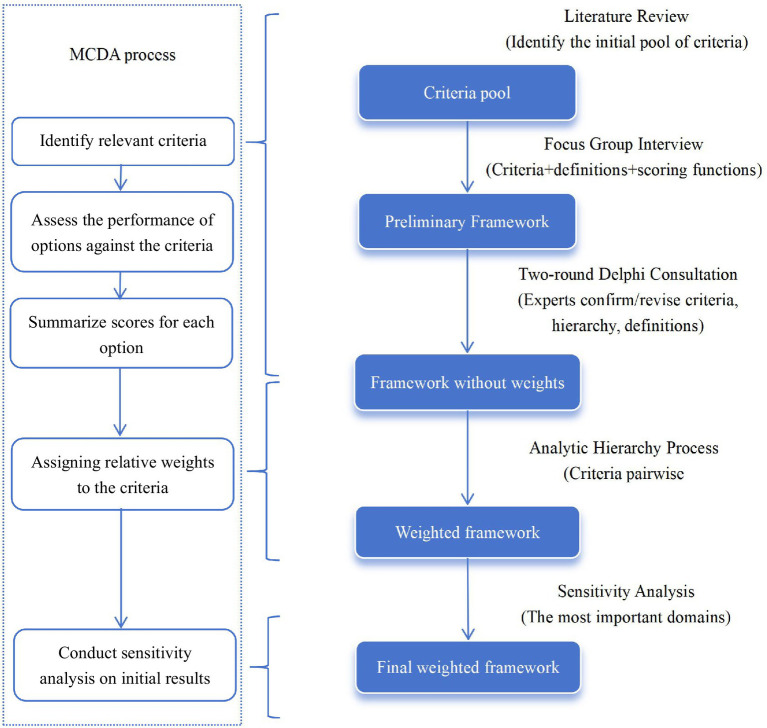
Flowchart of the comprehensive value assessment framework construction.

The comprehensive value assessment framework for RDN was created through literature review, focus group interviews, the Delphi method, and an Analytic Hierarchy Process (AHP) with the engagement of Chinese physicians and hospital administrators from different regional hospitals. A flowchart of the framework building process is shown in Figure 1. The literature review and focus group interviews inform the initial framework. The Delphi method then refines and validates the framework based on expert consensus. Finally, the AHP assigns weights to the validated framework to produce the final weighted value assessment framework. The AHP was not an independent step but rather a subsequent weighting exercise performed on the final framework confirmed by the Delphi process.

### Literature review

Relevant studies from a number of bibliographic databases, including China National Knowledge Infrastructure (CNKI), Wanfang Database, VIP Database, PubMed, and Wiley Online Library, were identified to serve as a reference for the framework and the pool of criteria. The search terms used were “medical device” OR “medical technology” combined with “value assessment” OR “Health Technology Assessment” OR “Multiple Criteria Decision Analysis” OR “multi-criteria decision analysis” OR “multicriteria decision analysis” in either the title or keywords field. The publication period was limited to January 2014 to December 2024. The titles and abstracts of the identified studies were reviewed to exclude the ineligible studies for full-text screening. The exclusion criteria were the following: (1) studies not discussing selection of medical devices, (2) studies not presenting clear decision criteria, (3) studies not discussing MCDA or not involving MCDA tools. The criteria and the scoring functions assessing the value of medical devices were extracted through full-text screening. Then, the identified criteria were clustered and stratified into domains and sub-criteria for further analysis.

### Focus group interview

A focus group was developed to formulate the preliminary framework based on the results of literature review, and the feedback from experts well experienced in value assessment, Chinese healthcare policies and the characteristics of RDN therapy for over 5 years, with intermediate professional title or above. Accordingly, two RDN therapy experts and three Health Technology Assessment (HTA) experts were selected to participate in the focus group interviews. With the informed consent of these participants, the interviews were recorded through written notes and audio recordings. Three rounds of interviews were conducted to make the set of criteria in correspondence with the properties required: completeness, non-redundancy, non-overlap, and preference independence (8). After multiple rounds of modifications, the preliminary comprehensive value assessment framework for RDN was formed.

### Delphi method

Two cardiologists with over 10 years’ experience in multiple RDN products and two non-clinical professionals in hospital procurement and health economics were invited to participate in the survey. The Delphi exercise in this study aimed at framework refinement (clarity, redundancy, and operability). A questionnaire was designed to assess the rationality of logic relationships between different criteria levels, the readability of each criterion, the redundancy among criteria, and the reliability of the scoring functions. Two rounds of questionnaires were distributed through email and one-to-one online interviews to achieve expert consensus on the criteria setting and the framework construction.

In the first round, experts were asked to provide a binary judgment on each criterion: “retain as is” “revise (please specify suggested changes)” or “delete,” as the primary goal was to elicit expert-informed content validation and refinement rather than statistical consensus thresholds. The research team reviewed all expert feedback and made decisions based on the following pre-specified rules: Retain without change: All experts agreed to “retain as is” or at least 75% of experts voted “retain as is” with no major objections in comments. Revise: Two or more experts suggested a specific modification, or one expert proposed a change that the research team judged as conceptually important and non-redundant. Delete: All experts voted “delete” or at least 50% voted “delete” with no strong opposing rationale, or the criterion was judged as overlapping with another existing criterion. Add new criterion: at least two experts proposed the same new criterion, or one expert proposed a clearly justified new criterion that filled an important gap. Based on the above rules, the team categorized all feedback, revised or deleted criteria accordingly, and added new ones where justified.

A second-round questionnaire was then developed, which included: (1) all criteria retained without change; (2) revised criteria presented alongside their original wording and a summary of expert suggestions; (3) newly proposed criteria. In the second round, experts were again asked to provide a final “retain/revise/delete” judgment, with the option to comment further. The process concluded when no further substantive changes were proposed by any expert.

All these experts who completed the previous round were also invited to participate in the subsequent round. Following the aggregated feedback from all the experts, items demonstrating a lack of consensus were incorporated into the second-round questionnaire for further assessment. The framework would be revised according to the comments once the consensus was reached (Consensus was defined as 75% agreement among all experts) (13).

### Analytical hierarchy process (AHP)

The AHP was implemented to determine the weight of each criterion, which mainly comprised three steps: (1) establish a hierarchical model (2) construct a pairwise comparison matrix and (3) evaluate the consistency and determine relative weights of criteria at each level (the consistency ratio (CR) < 0.1 indicates that consistency is acceptable). In the second step, the matrix consisted of all possible pairwise comparisons in the same hierarchy. The clinical and non-clinical experts were then invited to answer the pairwise comparison questions in a questionnaire by choosing a 5-point scale representing the intensity of their preferences between two criteria, ranging from “extreme unimportance” (ratio = 1) to “extreme importance” (ratio = 5). For a more detailed description of AHP methodology, it is referred to other researchers’ studies and publications (14, 15).

For clinical and non-clinical experts, the qualification requirements were: (1) hospital administrators or cardiology physicians from tertiary hospitals, (2) senior professionals with at least 10 years of healthcare experience, (3) decision-makers in hospital procurement. Yaahp 10.3 was used to analyze the data. The AHP involved all of the Delphi panel members as well as additional experts, resulting in a larger overall sample size for the AHP than for the Delphi process.

To assess the robustness of the derived criterion weights, a one-way sensitivity analysis was performed. The weights of the two most important domains were each varied by −10, 0 and +10% of their original values. For each adjustment, the weights of the other domains were proportionally adjusted so that all seven domain weights summed to 100%. The ranking of the first-level domains was examined across all variations to determine whether the top two positions remained stable. The analysis was implemented in Microsoft Excel.

## Results

### Literature review and focus group interviews

According to the literature review, a total of 787 publications were identified in the databases searched. After removing duplicates and reviewing the titles and abstracts, 17 studies and expert consensus that met our inclusion criteria were selected for full-text review (6, 16–32). Based on the literature review, a total of seven first-level, 18 second-level, and 33 third-level criteria were identified and incorporated into the criteria pool as the reference for the initial framework. The preliminary version of the framework specifically for RDN was then developed through focus group interviews, which was categorized into seven domains and three levels with detailed criteria, definitions, and scoring functions. At the first level, the seven domains are innovation, efficacy, safety, cost, qualification and suitability, patient value and management value. The second level criteria and third level sub-criteria or attributes are subdivided from the seven domains.

### Delphi consultation results

The Delphi consultation yielded a high degree of consensus among experts, with a 100% response rate in both rounds. All experts confirmed that the framework, its three-level hierarchy, and all criteria were understandable, mutually independent, and operational. Following the consensus of the two Delphi rounds, several main modifications were made to improve the conciseness, completeness and the rationality of the criteria. In the domain of efficacy, to optimize conciseness, multiple efficacy endpoints from RDN clinical trials were reclassified to two second-level criteria: short-term efficacy and long-term efficacy. The third-level criterion “alignment with reimbursement policy” was moved from the domain of innovation to the domain of management value. In addition, the definitions of six third-level attributes were refined referring to the domain of qualification and suitability, innovation, cost and management value. The updated version was confirmed, including three levels (first level: seven domains; second level: 16 criteria; third level: 30 sub-criteria or attributes), as shown in Table 1.

**Table 1:** Comprehensive value assessment framework for RDN therapy.

First-level criteria	Weight	Second-level criteria	Weight	Third-level Sub-criteria	Weight
Qualification and suitability	16.84%	Qualification	4.67%	NMPA regulatory approval	2.96%
				Ethical compliance	1.71%
		Technical suitability	6.76%	Technological maturity	6.76%
		Procedure appropriateness	5.41%	Ease of use	2.13%
				Learning curve	1.48%
				Technical support capacity	1.80%
Innovation	10.65%	Breakthrough device designation	7.22%	International authorization	4.13%
				Domestic authorization	3.09%
		Therapeutic advances	3.43%	Adaptability to complex anatomical structures	3.43%
Safety	21.83%	Procedure-related adverse events	21.83%	Intraoperative adverse events	6.99%
				Short-term postoperative adverse events	6.39%
				Long-term postoperative adverse events	8.45%
Efficacy	23.26%	Blood pressure reduction	23.26%	Short-term blood pressure reduction	5.91%
				Long-term blood pressure reduction	17.35%
Cost	6.01%	Direct medical costs	2.44%	Procedure cost	1.22%
				Cost of intraoperative adverse events	0.78%
				Cost savings from reduced medications	0.44%
		Costs from a hospital perspective	1.76%	Labor costs	1.18%
				Facility costs	0.58%
		Costs from a societal perspective	1.81%	Productivity losses and family burden	1.81%
Patient value	15.18%	Medication adherence	6.31%	Stable medication usage rate	6.31%
		QoL	8.87%	Short-term QoL improvement	4.20%
				Long-term QoL improvement	4.66%
Management value	6.22%	Accessibility	0.97%	Hospital penetration	0.41%
				Supply chain reliability	0.56%
		Quality stability	2.47%	Durability	1.76%
				Quality management system	0.71%
		Brand reputation	1.45%	Technical reputation	0.85%
				Service reputation	0.60%
		Compatibility	1.33%	Alignment with reimbursement policy	1.33%

NMPA, National Medical Products Administration; Qol, Quality of Life.

The domain order follows a hierarchical progressive logic based on hospital access considerations for innovative medical services and devices. Qualification and suitability are positioned as the basic prerequisite for access, followed by innovation—since only innovative products warrant comprehensive value assessment. The remaining domains are then ordered by their assessed importance: safety, efficacy, cost, patient value, and management value.

### Criteria revision and refinement

The proposed framework comprises seven domains, each operationalized through a set of second-level criteria and third-level sub-criteria tailored to the specific characteristics of RDN therapy. These domains collectively address the key considerations in hospital procurement decisions: regulatory and operational readiness (Qualification and Suitability), technological advancement (Innovation), clinical safety and efficacy (Safety and Efficacy), economic impact from multiple stakeholder perspectives (Cost), patient-centered outcomes (Patient Value), and institutional management priorities (Management Value). The following sections detail the rationale and composition of each domain.

#### Qualification and suitability

This domain was employed to assess the qualifications, the ethics and the suitability of application of different RDN products in hospitals. Three second-level criteria and six third-level attributes were customized to the assessment of RDN product regulatory clearances, ethical compliance, technology design, domestic and international clinical acceptance, learning curve and technical support capacity.

#### Innovation

To measure the degree of innovation for different RDN products, the innovation domain was comprised of two second-level criteria and three third-level attributes. These criteria focused on reviewing the innovation designations authorized by domestic and international regulatory authorities and evaluating the innovative designs by improving their clinical adaptability to accommodate patients with different anatomical features of renal artery.

#### Safety

Hospital administrators and physicians gave significant consideration to safety. In order to evaluate intraoperative, short-term, and long-term safety, one second-level criterion and three third-level sub-criteria were established to specify RDN procedure-related adverse events. In accordance with the multiple well-designed clinical trials of RDN (11, 12, 29, 33–38) and the experts’ consensus, 6 months were defined as the cutoff point between short-term and long-term safety.

#### Efficacy

Regarding hypertension as a chronic disease requiring lifelong treatment, both short-term and long-term antihypertensive efficacy of RDN products should be considered. One second-level criterion and two third-level sub-criteria were implemented in the efficacy domain. Currently, different primary and secondary endpoints were performed to estimate the efficacy of RDN in various clinical trials (11, 12, 33–39). In light of the results from focus group interviews and the Delphi process, the outcomes of office blood pressure reduction, night-time ambulatory blood pressure reduction, and time in target blood pressure range were utilized to assess the short-term and long-term efficacy of RDN.

#### Cost

The cost is the domain that most frequently appeared in the MCDA studies (6). With respect to hospital procurement, costs from different stakeholder perspectives are analyzed in the decision-making process. Three second-level criteria and six third-level attributes guided the cost assessment, covering the direct and indirect costs from the perspective of patients, hospitals and society. Economic evaluation was not employed as a criterion in this domain to prevent the overlap with the domain of patient value.

#### Patient value

Symptoms of hypertension and taking numerous medications substantially impact patients’ Quality of Life. Effective RDN treatment contributes to the reduction in blood pressure and medication requirements, which could lead to the relief of symptoms and the improvement of medication adherence (40–42). Thus, the patient value domain was adopted to evaluate the influence of different RDN products on patients’ health status and Quality of Life. In this domain, there were two criteria at the second level and three sub-criteria at the third level to assess the effects of RDN products on patients’ medication adherence, short-term and long-term Quality of Life (QoL), respectively.

#### Management value

From the perspective of hospital management value, three second-level criteria and seven third-level sub-criteria were incorporated into this domain. As for the utilization of innovative technology, hospitals emphasized the product quality, accessibility and brand reputation. In terms of hospital procurement, Chinese hospitals strictly followed the market access requirements for innovative technologies to ensure compliance with healthcare and reimbursement regulations. In this case, the “alignment with the reimbursement policy” was set as a third-level criteria in the management value domain.

### Analytic Hierarchy Process (AHP)

Nineteen experts from public hospitals in various geographic regions answered the pairwise comparison questionnaire, including 11 cardiologists and eight non-clinical professionals. All of them have more than 10 years’ working experience in Chinese tertiary hospitals. 94.74% of them held senior titles and 5.26% of them obtained associate senior titles. Among eight non-clinical professionals, four of them are from hospital procurement department, three of them are from health insurance department and one is from the medical service department. Details are shown in Table 2.

**Table 2:** Distribution of AHP expert professional titles and years of experience.

Professional title/years of experience	Count	Percentage
Professional title		
Junior	0	0.00%
Intermediate	0	0.00%
Associate senior	1	5.26%
Senior	18	94.74%
Total	19	
Years of experience		
≤5 years	0	0.00%
5–10 years	0	0.00%
≥10 years	19	100.00%
Total	19	

The weighting results were summarized in Table 1. All criteria passed the consistency test. The detailed criteria definition, scoring functions and the results of consistency tests are in Supplementary Tables S1, S2. Among the seven domains, safety and efficacy carried the highest weights, which were 21.83 and 23.26% respectively, while cost and management value had the lowest weights, which were 6.01 and 6.22%, respectively.

One-way sensitivity analysis of the criterion weights shows that when the Efficacy or Safety weight is reduced by 10%, Safety becomes the highest-ranked domain, but the top two positions are always occupied by Efficacy and Safety (in either order). Thus, the relative dominance of clinical outcome domains (Efficacy + Safety) is highly robust to changes in the Efficacy weight.

## Discussion

In China, a growing body of scholars is exploring the application of MCDA to support healthcare policy decisions and medical device management. Various researchers have developed a series of value assessment frameworks aimed at guiding procurement and reimbursement decisions for medical devices (17, 30–32). However, these frameworks either propose a general assessment approach applicable to all devices without distinguishing between decision-making scenarios, or they are tailored to specific devices (such as surgical staplers), and therefore not fully suitable for innovative technologies like RDN (16–21, 30–32). To our knowledge, this study presents the first comprehensive value assessment framework specifically designed for RDN devices to support hospital-level decision-making in China. The framework is customized to reflect the distinctive characteristics of RDN therapy and incorporates the perspectives of all stakeholders involved in hospital decision-making process. Most importantly, it offers a practical value assessment tool for hospitals to support RDN product selection, featuring clearly defined criteria, assigned weights and scoring functions.

### Comparison of criteria: similarities and differences across studies

To align with the MCDA guideline, a three-level framework was established to ensure completeness, non-redundancy, non-overlap, and preference independence (8). Among the seven defined domains, qualifications and suitability, efficacy, safety, patient value and cost are the common dimensions founded in conventional value assessment frameworks (16–29). In this framework, the criteria within these domains were specifically designed and refined to capture the characteristics of hypertension disease and RDN therapy.

Regarding the cost domain, cost-effectiveness-particularly the incremental cost-effectiveness ratio (ICER)-was not included as a third-level criterion, which is consistent with the criteria settings adopted in several previous studies (16, 32). In addition, Chen’s study also considered “ICER” to be of relatively low importance in the selection of criteria for hospital-level decision making (31). Two main reasons may explain this choice. First, direct costs, indirect costs and patients’ Quality of Life, which are key components of cost-effectiveness analyses, are already encompassed within cost and patient value domains. Including cost-effectiveness separately would result in redundant evaluation and disproportionately increase the weight of these aspects. Second, the framework is intended for comparing different RDN devices, and no published economic evaluations directly comparing RDN devices are currently available.

The innovation domain which frequently appears in the general assessment framework for evaluating resource allocation across different therapies and populations, is less common in frameworks designed for products within the same therapeutic category (18–21, 32, 44). Nevertheless, it was included in this RDN-specific framework due to the following considerations. Unlike mature, widely familiar therapies, RDN is a disruptive therapy that has only been introduced in the Chinese market for about a year. Moreover, all four currently launched RDN products have received innovation designations from NMPA. However, actual clinical experience with these devices remains limited, and their designs, features, and functions vary significantly. From a hospital perspective, relying solely on regulatory innovation designations is insufficient for scientifically evaluating “medical device innovation” and making informed procurement decisions. Therefore, the second-level criterion “therapeutic advances” was established to provide a clearer, more substantive assessment of innovation for transformative devices, aligning with the approach taken in Chen et al.’s study (31).

The management value domain is incorporated in our framework, consistent with other studies focused on hospital-level decision-making (17, 30, 31). Notably, this study introduces a unique second-level criterion- “compatibility”-alongside accessibility, quality and brand reputation. In China, public hospitals often face policy barriers when adopting device-based therapies, especially disruptive technologies. Common issues include the lack of compatible charge codes^1^, Diagnosis-Related Groups add-on payments, or reimbursement mechanisms for new medical device technologies, which can hinder the introduction of innovative therapies. This criterion reflects the potential operational and financial impacts of adopting a new medical device within the context of government-mandated cost-control requirements for hospitals.

### Comparison of criteria weights: similarities and differences across studies and therapeutic areas

In the weight distribution derived from this study, clinical-related value—encompassing efficacy, safety, and qualification and suitability—received the highest weights, a pattern consistent with several prior studies (16, 17, 19, 20). This also reflects the current development stage of RDN therapy in China. With four RDN products having recently entered the market with limited applications in public hospitals. Considering that not all RDN products’ performance in the real world has been well-recognized at the time of the study, both physicians and hospital administrators agreed that the clinical-related value of RDN products should be ensured and prioritized.

It is worth mentioning that this study has assigned a relatively high weight to the innovation domain (10.65%), and the lowest weight to the cost domain (6.01%), which contrasts with common perception of China’s cost-control-oriented health policies and differs from the weight patterns reported in some other studies (30, 32, 45). For example, in Xu et al.’s (32) study, innovation received nearly the lowest weight (2.54%), while cost ranked second highest among the six criteria domains. Several factors may explain this divergence. Xu et al.’s (32) framework was designed from a budget allocation perspective for general implantable device reimbursement decisions, where most medical device innovations are incremental improvements rather than breakthroughs; thus, cost considerations naturally gained prominence. In contrast, the present framework is specifically tailored for RDN therapy which is viewed as a breakthrough catheter-based technology for hypertension treatment. Also, it is intended for hospital procurement decisions. The higher weight placed on innovation indicates that, in this context, hospital management prioritizes enhancing clinical capabilities and delivering advanced patient care. Meanwhile, the low weight assigned to cost suggests that, despite policy and budgetary constraints, hospitals are willing to allocate resources to innovative medical devices that demonstrate superior clinical value, high suitability, and breakthrough potential.

### Applications and policy implications of this framework

This study presents a preliminary comprehensive value assessment framework for RDN therapy, which can serve as a pilot for the evaluation of innovative medical devices to support hospital-level procurement decision-making in China. Given the heterogeneity of medical device characteristics, decision-making objectives, and institutional contexts, there is no “one-size-fits-all” MCDA framework applicable across all application scenarios. Instead, scenario-based dynamic adjustment of criteria weights is both necessary and consistent with international MCDA good practices and decision-oriented HTA principles (8, 28, 44).

From an application perspective, within the hospital procurement context, the framework allows for post-adoption dynamic reassessment by hospitals through modifying the relative importance of value domains while maintaining a stable core criteria structure. As clinical experience accumulates and additional evidence on long-term effectiveness, safety, learning curves, and operational performance becomes available, hospitals may iteratively reapply the framework to support continued use evaluation, product re-selection, and procurement portfolio adjustment of RDN devices. In such reassessment processes, greater emphasis may gradually shift from innovation-related criteria toward long-term efficacy, safety, management value, and real-world operational performance, reflecting the transition from early adoption to routine clinical use. Operationally, such adjustments can be implemented by re-running the AHP weighting process with hospital-based stakeholder panels or by applying predefined weight reference ranges informed by decision objectives, as recommended in prior MCDA and HTA studies (7, 9, 44) (Supplementary Table S3). We also performed an initial dry-run application by inviting experts to apply the framework to the currently approved RDN devices to test feasibility; further validation using real-world hospital procurement cases is warranted.

Beyond the primary hospital procurement scenario, this framework also provides insights for broader decision-making contexts. For example, in medical insurance reimbursement or coverage deliberations, cost-related criteria and long-term effectiveness may warrant greater emphasis, aligning with payer objectives of budget impact control and population-level health outcomes. At a regional health planning level (e.g., regional service capacity building), increased attention to innovation and patient value may support equitable access to breakthrough technologies and address unmet clinical needs. These potential extensions illustrate the flexibility of the framework, although further scenario-specific validation would be required (Supplementary Table S3).

This framework also has important implications for the development of China’s national medical device HTA system. Currently, value assessment mechanisms for medical devices remain fragmented and less mature compared with those for pharmaceuticals, particularly for innovative and rapidly evolving technologies. This study demonstrates the feasibility of using tailored, device-specific MCDA frameworks to complement conventional HTA approaches by explicitly incorporating innovation characteristics, learning curves, organizational impact, and policy compatibility. Regulatory authorities such as the NMPA and the National Healthcare Security Administration (NHSA) could consider promoting similar scenario-oriented, multi-criteria value assessment frameworks for other innovative medical devices, thereby supporting more transparent regulatory evaluation, reimbursement deliberation, and technology adoption decisions. Such an approach would be consistent with China’s ongoing health system reforms emphasizing value-based healthcare, rational resource allocation, and high-quality development of public hospitals (43).

### Limitations

This study has several limitations:

First, most of the scoring functions rely on qualitative judgments. As RDN is a newly introduced therapy, clinical data and real-word evidence across products remain limited, making fully quantitative scoring functions impractical at this stage. As more evidence emerges, the scoring functions and thresholds should be periodically refined to improve objectivity and reproducibility.

Second, although the expert panel included both clinical and non-clinical hospital decision-makers, the sample size was relatively small, which may limit representativeness. In addition, because RDN is currently performed mainly in tertiary hospitals in China, it was not feasible to recruit experts from primary/secondary hospitals during this study period. Future studies should expand expert representation across a wider range of institutions and geographic regions, including regional medical centers in central and western China, as RDN adoption increases.

Third, the weighting exercise reflects the preferences of hospital decision-makers, which is consistent with the intended application scenario (hospital procurement). Patient representatives were not included as formal decision-makers in the current study, reflecting real-world hospital procurement practices. Nevertheless, to strengthen patient-centeredness of the framework, future work could incorporate patient perspectives through complementary approaches (e.g., patient surveys/interviews, discrete choice experiments, and patient-reported outcomes evidence) to inform relevant criteria definitions, scoring functions, or sensitivity analyses.

Finally, we have not yet validated the framework using real-world hospital procurement cases or assessed concordance with actual procurement decisions; such multi-center pilot validation will be needed as RDN adoption increases.

## Conclusion

The comprehensive value assessment framework for RDN therapy was developed through a MCDA approach with scientific evaluation from hospital clinical and non-clinical experts. It provides a transparent and practical reference to inform the selection and adoption of RDN devices in Chinese hospital settings. More broadly, it may also serve as a methodological reference for developing tailored assessment frameworks for other innovative medical devices across different decision-making contexts, with scenario-specific adaptation as needed.

## Data Availability

The raw data supporting the conclusions of this article will be made available by the authors, without undue reservation.

## Footnotes

1 Charge code is a unique alphanumeric identifier assigned to a specific medical service or procedure, used for billing and reimbursement purposes in healthcare management.

## Author’s note

The primarily version of this study was oral presented for discussion and the poster of this study was presented on conference (Great Wall International Congress of Cardiology (GW-ICC) 2025).
