# Supplementary material for: Construction of a comprehensive value assessment framework for renal denervation therapy: a decision-support tool for Chinese hospital management
Source: Front Public Health. 2026 Jun 5;14:1801333. doi: 10.3389/fpubh.2026.1801333 (PMC13279309; doi:10.3389/fpubh.2026.1801333)
Supplement: Supplementary file 1 [file Data_Sheet_1.DOCX]

**Supplementary Table S1 Comprehensive Value Assessment Framework for RDN Therapy**

| First-level Criteria | Second-level Criteria | Third-level Sub-Criteria | Criteria Definition | Scoring standards (0-10, with 10 being the highest score) |
| --- | --- | --- | --- | --- |
| Qualification and Suitability | Qualification | NMPA Regulatory approval | Verify that the product explicitly identifies the corresponding medical technology and possesses NMPA approval. | **10 points:** The product has a clearly defined medical technology name matching its consumables and holds regulatory approval from NMPA. **0 points:** The product does not have NMPA regulatory approval. |
|  |  | Ethical Compliance | Assess whether the product complies with the four ethical principles: non-maleficence, beneficence, respect, and justice**.** | **10 points**: The product adheres to the four principles of medical ethics  **0 points**: The product violates the four principles of medical ethics |
|  | Technical Suitability | Technological Maturity | Appraise the product’s technological maturity by considering time since market introduction (domestic and international), cumulative procedure volume, and adequacy of supporting clinical evidence. | **10 points**: Marketed for >3 years and preceding most comparators, the device has been deployed in >1,000 procedures worldwide; its safety and efficacy are substantiated by high-level clinical evidence.  **0 points**: Commercialized only recently, the device has yet to be employed domestically and lacks supporting clinical-trial data on safety or efficacy. |
|  | Procedure Appropriateness | Ease of Use | Assess device design compatibility with standard interventional workflows: arterial access route (radial vs femoral), guide catheters, guidewires, the diameter of the catheter, and the equipment intelligence level. | **10 points**: The product's arterial access route aligns with clinician’s operational habits. It offers matched guide catheters and guidewires. The diameter of the catheter is perfectly appropriate, providing excellent operational convenience. In addition, the equipment is highly intelligent.  **0 points**: The product's arterial access route does not align with clinician’s operational habits. It lacks matched guide catheters and guidewires. The diameter of the catheter is inappropriate with poor operational convenience. The equipment is not intelligent at all. |
|  |  | Learning Curve | Assess the complexity of the procedure, simplicity of workflow, and time required for clinicians to master the technology. | **10 points**: The procedure is extremely simple, with a very short learning curve. **0 points**: The procedure is extremely complex, with a very long learning curve. |
|  |  | Technical Support Capacity | Evaluate the professionalism and comprehensiveness of training programs from different manufacturers. | **10 points**: There is a professional team with a comprehensive training plan for preoperative training and clinical center activation, as well as technical support. **0 points**: The manufacturer does not offer any professional training or technical support. |
| Innovation | Breakthrough Device Designation | International Authorization | Whether the product has received the FDA Breakthrough Device Designation in the U.S. | **10 points**: The product has received FDA Breakthrough Device designation in the U.S. **0 points**: The product does not have FDA Breakthrough Device designation. |
|  |  | Domestic Authorization | Whether the product has passed China’s NMPA green channel for Innovative Medical Device Special Approval. | **10 points**: The product has passed NMPA’s green channel for innovative medical device special approval . **0 points**: The product does not pass NMPA's green channel innovative medical device special approval. |
|  | Therapeutic Advances | Applicability to complex anatomical structure | Assess the applicability to diverse renal artery anatomies (e.g., small branches, tortuous vessels, accessory renal arteries). | **10 points**: Extremely suitable. **0 points**: Extremely unsuitable. |
| Safety | Procedure-Related Adverse Events | Intraoperative Adverse Events | Incidence of adverse events during the procedure. | **10 points**: No intraoperative complications (0%)  **8-9 points**: Minor complications (e.g., transient renal artery spasm) requiring no intervention  **6-7 points**: Mild complications requiring conservative management  **4-5 points**: Moderate complications requiring prolonged observation  **2-3 points**: Severe complications (e.g., renal artery dissection or perforation) requiring endovascular intervention **0-1 points**: Fatal or life-threatening complication |
|  |  | Short-Term Postoperative Adverse Events | Incidence of adverse events within six months. | **10 points**: No adverse events, or self-limiting mild events <1%  **8-9 points**: The adverse event rate 1-3%  **6-7 points**: 3-7%  **4-5 points**: 7-15%  **2-3** **points**: 15-30% **0-1 points**: ≥30%. |
|  |  | Long-Term Postoperative Adverse Events | Incidence of adverse events ＞ six months post-procedure. | **10 points**: No long-term adverse events, or <0.5%/year  **8-9 points**: The adverse event rate 0.5% -1%/year  **6-7 points**: 1% -3%/year  **4-5 points**: 3% -5%/year  **2-3 points**: 5% -7%/year **0-1 points**: ≥7.0%/year |
| Efficacy | Blood Pressure Reduction | Short-Term Blood Pressure Reduction | Reduction of Office blood pressure and night-time ambulatory blood pressure, and time in target blood pressure range within six months. | **10 points**:Office SBP Reduction ≥20mmHg; Night-Time Ambulatory SBP Reduction ≥12mmHg; Time in Target BP Range 80%  **8-9 points**: Office SBP Reduction 14-20 mmHg; Night-Time Ambulatory SBP Reduction 8-12 mmHg; Time in Target BP Range 60%-80%  **6-7 points**: Office SBP Reduction 8-14 mmHg; Night-Time Ambulatory SBP Reduction 5-8 mmHg; Time in Target BP Range 40%-60%  **4-5 points**: Office SBP Reduction 3-8 mmHg; Night-Time Ambulatory SBP Reduction 3-5 mmHg; Time in Target BP Range 20%-40%  **2-3 points**: Office SBP Reduction 0-3 mmHg; Night-Time Ambulatory SBP Reduction 1-3 mmHg; Time in Target BP Range 5%-20%  **0-1 points**: Office SBP Reduction <0 mmHg (increase); Night-Time Ambulatory SBP Reduction <1 mmHg; Time in Target BP Range <5% |
|  |  | Long-Term Blood Pressure Reduction | Office blood pressure, night-time ambulatory blood pressure, and time in target blood pressure range after six months or longer. | **10 points: Office SBP Reduction ≥18 mmHg; Night-Time Ambulatory SBP Reduction ≥10 mmHg; Time in Target BP Range ≥75%**  **8-9 points: Office SBP Reduction 12-18 mmHg; Night-Time Ambulatory SBP Reduction 7-10 mmHg; Time in Target BP Range 55%-75%**  **6-7 points: Office SBP Reduction 6-12 mmHg; Night-Time Ambulatory SBP Reduction 4-7 mmHg; Time in Target BP Range 35%-55%**  **4-5 points: Office SBP Reduction 2-6 mmHg; Night-Time Ambulatory SBP Reduction 2-4 mmHg; Time in Target BP Range 15%-35%**  **2-3 points: Office SBP Reduction 0-2 mmHg; Night-Time Ambulatory SBP Reduction 0-2 mmHg; Time in Target BP Range 5%-15%**  **0-1 points: Office SBP Reduction <0 mmHg (increase); Night-Time Ambulatory SBP Reduction <0 mmHg (increase); Time in Target BP Range <5%** |
| Cost | Direct Medical Costs | RDN Procedure Cost | Costs of the RDN product, associated consumables, and other intraoperative expenses. | **10 points**: Compared to similar products, the total cost of the procedure is significantly reduced. **0 points**: Compared to similar products, the total cost of the procedure is significantly increased. |
|  |  | Cost of intraoperative Adverse Events | Costs of treating intraoperative adverse events. | **10 points**: Compared to similar products, the cost of managing intraoperative adverse events is significantly reduced. **0 points**: Compared to similar products, the cost of managing intraoperative adverse events is significantly increased. |
|  |  | Cost Savings from Reduced Medication Use | Savings from reduced antihypertensive medication use. | **10 points**: Compared to similar products, this product significantly reduces the cost of antihypertensive drugs. **0 points**: Compared to similar products, this product increases the cost of antihypertensive drugs. |
|  | **Costs From a Hospital Perspective** | Labor Costs | Number of clinicians and nurses required and average procedure duration. | **10 points**: Compared to similar products, this product significantly reduces the number of medical staff required for RDN procedure and shortens the average procedure time. **0 points**: Compared to similar products, this product significantly increases the number of medical staff required for RDN procedure and lengthens the average procedure time. |
|  |  | Facility Costs | The RDN equipment (including depreciation) costs. | **10 points**: Compared to similar products, the cost of the RDN equipment is significantly lower. **0 points**: Compared to similar products, the cost of the RDN equipment is significantly higher. |
|  | **Costs From a Societal Perspective** | Productivity Loss and Family Burden | Loss of productivity due to hospitalization/recovery time and family caregiving burden. | **10 points**: Compared to similar products, this product significantly shortens length of stay in hospital and recovery time. **0 points**: Compared to similar products, this product increases length of stay in hospital and recovery time. |
| Patient Value | Medication Adherence | Stable Medication Usage Rate | improvement in drug adherence due to reduction in antihypertensive medication types/quantities | **10 points**: This product significantly reduces antihypertensive drug use (types and quantities) and improves medication adherence. **0 points**: This product increases antihypertensive drug use (types and quantities) and decreases medication adherence. |
|  | **QOL** | Short-Term QOL Improvement | Postoperative (within six months) improvement in QoL scores by scales or estimated QoL enhancement. | **10 points**: Short-term QOL is significantly improved. **0 points**: Short-term QOL is significantly reduced. |
|  |  | Long-Term QOL Improvement | Improvement in QoL scores by scales or estimated QoL enhancement after 6 months or longer. | **10 points**: Patient QOL is significantly improved after six months.  **0 points**: Patient QOL is reduced after six months. |
| Management Value | Accessibility | Hospital Penetration | Number of public hospitals covered in China. | **10 points**: The product is available in 10 or more public hospitals nationwide. **0 points**: The product is not available in any public hospitals nationwide. |
|  |  | Supply Chain Reliability | Manufacturer’s production capacity, equipment, and staffing to ensure stable and timely supply. | **10 points**: The manufacturer has high production capacity, sufficient personnel, plant and machinery, which responds promptly. **0 points**: The manufacturer has low production capacity, insufficient personnel, plant and machinery, which responds slowly. |
|  | Quality Stability | Durability | Product durability (e.g., resistance to deformation, need for intraoperative catheter replacement, equipment trouble rate). | **10 points**: The product is durable, resistant to deformation, does not require catheter replacement during RDN procedure, and has a low equipment trouble rate. **0 points**: The product is not durable, prone to deformation, requires catheter replacement during RDN procedure, and has a high equipment trouble rate. |
|  |  | Quality Management System | Product and manufacturer certifications (e.g., ISO, GMP). | **10 points**: Certified by the Medical Device Quality Management System. **0 points**: Not certified. |
|  | Brand Reputation | Technical Reputation | Recognition of the manufacturer’s technical capability | **10 points**: The manufacturer has strong technical advantages and academic expertise in interventional therapy for hypertension and regularly organizes academic activities. **0 points**: The manufacturer lacks technical advantages and academic expertise in interventional therapy for hypertension and has not organized academic activities. |
|  |  | Service Reputation | Recognition of the manufacturer’s service capabilities. | **10 points**: The manufacturer has excellent qualifications and a strong reputation for service capability in the industry. **0 points**: The manufacturer has poor qualifications and a weak reputation for service capability in the industry. |
|  | Compatibility | Alignment with Reimbursement Policy | Whether the product improves the CMI; whether it has a clear DRG/DIP grouping pathway; whether it has charge codes for consumables/procedure, and whether it can be reimbursed by public health insurance | **10 points**: The product improves the CMI, has a clear DRG/DIP grouping pathway, and both the procedure and consumables are chargeable and covered by public health insurance. **0 points**: The product decreases the CMI, lacks a clear DRG/DIP grouping pathway, and neither the procedure nor consumables are chargeable or covered by public health insurance. |

RDN: renal denervation NMPA: National Medical Products Administration, FDA: U.S. Food and Drug Administration, QoL: quality of life, GMP: Good Manufacturing Practice of Medical Products, CMI: case-mix index, DRG: diagnosis related group, DIP: diagnosis-intervention pocket

Real-world evidence and multi-centre RDN registries should be used for annual updates as more data become available.

For products with no clinical evidence or insufficient evidence reporting, a neutral score of 5 should be assigned.

**Supplementary Table S2 The Results of Consistency Tests**

| **First-level Criteria** | **Weight** | **Consistency Test** | **Second-level Criteria** | **Weight** | **Consistency Test** | **Third-level Sub-Criteria** | **Weight** | **Consistency Test** |
| --- | --- | --- | --- | --- | --- | --- | --- | --- |
| **Qualification and Suitability** | 16.84% | **CR=0.021** **Pass** | Qualification | 27.74% | CR=0.000 Pass | NMPA Regulatory Approval | 63.37% | - |
|  |  |  |  |  |  | Ethical Compliance | 36.63% | - |
|  |  |  | Technical Suitability | 40.12% | - | Technological Maturity | 100.00% | - |
|  |  |  | Procedure Appropriateness | 32.14% | CR=0.002 Pass | Ease of Use | 39.41% | - |
|  |  |  |  |  |  | Learning Curve | 27.33% | - |
|  |  |  |  |  |  | Technical Support Capacity | 33.26% | - |
| **Innovation** | 10.65% | - | Breakthrough Device Designation | 67.83% | - | International Authorization | 57.21% | - |
|  |  |  |  |  |  | Domestic Authorization | 42.79% | - |
|  |  |  | Therapeutic Advances | 32.17% | - | Applicability to complex anatomical structure | 100.00% | - |
| **Safety** | 21.83% | - | Procedure-Related Adverse Events | 100.00% | CR=0.000 Pass | Intraoperative Adverse Events | 32.02% | - |
|  |  |  |  |  |  | Short-Term Postoperative Adverse Events | 29.28% | - |
|  |  |  |  |  |  | Long-Term Postoperative Adverse Events | 38.70% | - |
| **Efficacy** | 23.26% | - | Blood Pressure Reduction | 100.00% | - | Short-Term Blood Pressure Reduction | 25.39% | - |
|  |  |  |  |  |  | Long-Term Blood Pressure Reduction | 74.61% | - |
| **Cost** | 6.01% | CR=0.012 Pass | Direct Medical Costs | 40.53% | CR=0.015 Pass | RDN Procedure Cost | 50.05% | - |
|  |  |  |  |  |  | Cost of intraoperative adverse events | 31.92% | - |
|  |  |  |  |  |  | Cost Savings from Reduced Medication Use | 18.03% | - |
|  |  |  | Hospital Perspective Costs | 29.31% | - | Labor Costs | 67.24% | - |
|  |  |  |  |  |  | Facility Costs | 32.76% | - |
|  |  |  | Societal Costs | 30.16% | - | Productivity Loss and Family Burden | 100.00% | - |
| **Patient Value** | 15.18% | - | Medication Adherence | 41.6% | - | Stable Medication Usage Rate | 100.00% | - |
|  |  |  | QoL | 58.4% | - | Short-Term QoL Improvement | 47.42% | - |
|  |  |  |  |  |  | Long-Term QoL Improvement | 52.58% | - |
| Management Value | 6.22% | CR=0.002 Pass | Accessibility | 15.56% | - | Hospital Penetration | 42.07% | - |
|  |  |  |  |  |  | Supply Chain Reliability | 57.93% | - |
|  |  |  | Quality Stability | 39.72% | - | Durability | 71.15% | - |
|  |  |  |  |  |  | Quality Management System | 28.85% | - |
|  |  |  | Brand Reputation | 23.32% | - | Technical Reputation | 58.58% | - |
|  |  |  |  |  |  | Service Reputation | 41.42% | - |
|  |  |  | Compatibility | 21.39% | - | Alignment with Reimbursement Policy | 100.00% | - |

CR: consistency ratio; QoL: quality of life

# **Supplementary Table S3 Scenario-specific reference ranges for rebalancing first-level domain weights (illustrative)**

| **First-level Criteria** | **Baseline** Hospital procurement | **Scenario A:** Hospital post-adoption dynamic reassessment (continued use / re-selection) | **Scenario B:** Reimbursement/coverage deliberation (payer perspective) | **Scenario C:** Regional health planning / access decision (system perspective) |
| --- | --- | --- | --- | --- |
| **Efficacy** | 23.26% | 18–30% | 15–28% | 12–26% |
| **Safety** | 21.83% | 16–28% | 14–26% | 12–24% |
| **Qualification & suitability** | 16.84% | 10–22% | 8–20% | 8–20% |
| **Patient value** | 15.18% | 10–22% | 8–22% | 14–30% |
| **Innovation** | 10.65% | 3–15% | 2–12% | 8–25% |
| **Management value** | 6.22% | 5–18% | 2–12% | 4–16% |
| **Cost** | 6.01% | 3–15% | 10–30% | 6–20% |

Baseline weights were derived from the AHP exercise in this study for hospital procurement at adoption.

1. The ranges are illustrative reference ranges intended to support practical rebalancing discussions; they are not prescriptive and do not represent re-estimated weights. Final weights should be confirmed via scenario-specific stakeholder elicitation (e.g., repeated AHP or structured expert reassessment) as recommended in MCDA good practices
2. Scenario rationale:

- Hospital post-adoption reassessment: as experience accumulates, emphasis may shift from early “innovation” signals to long-term outcomes, safety, and operational/management performance in real-world use.
- Reimbursement/coverage: greater emphasis on cost and long-term effectiveness reflecting budget impact and population-level health objectives.
- Regional health planning/access: increased emphasis on innovation, patient value, and implementation feasibility to support unmet need and equitable access, while maintaining minimum clinical and safety requirements.

# **Supplementary: RDN Therapy Comprehensive Value Assessment Framework – Operational Manual**

## **1. Purpose and Scope**

This manual provides standardized procedures for applying the RDN value assessment framework to support hospital procurement decisions. The framework evaluates four approved RDN products across seven domains: Qualification and Suitability, Innovation, Safety, Efficacy, Cost, Patient Value, and Management Value.

**2. Assessment Process Overview The assessment follows a five-step process:**

| Step | Activity | Responsible Party |
| --- | --- | --- |
| 1 | Data collection for each RDN product | Clinical & Procurement teams |
| 2 | Scoring each third‑level sub‑criterion | Assigned experts (see Section 4) |
| 3 | Weighted score calculation | Analyst |
| 4 | Sensitivity check (optional) | Analyst |
| 5 | Final ranking and procurement recommendation | Multi‑disciplinary committee |

1. **Scoring System**

**3.1 Scoring Scale Each third-level sub-criterion is scored on a 0-10 scale (integer values)**

| Score | Descriptor | Interpretation |
| --- | --- | --- |
| 10 | Perfect | Flawless standard |
| 8-9 | Excellent | Far exceeds acceptable standard |
| 6-7 | Good | Meets and sometimes exceeds standard |
| 4-5 | Acceptable | Meets minimum standard |
| 2-3 | Poor | Below acceptable standard, but not unacceptable |
| 0-1 | Unacceptable | Fails to meet basic requirements |

**3.2 Quantitative vs. Qualitative Criteria**

Quantitative criteria(e.g., blood pressure reduction, adverse event rates): Use the predefined 0-10 point scoring functions provided in the Value Assessment Framework, Scoring standards.

Qualitative criteria(e.g., ethical compliance, brand reputation): Use the scale with anchor descriptors provided in the Value Assessment Framework, Scoring standards.

## **4. Role Assignment and Responsibility**

Assessment requires input from clinical experts (cardiologists, interventionalists) and non-clinical experts (hospital procurement, health economics, administration). Their distinct perspectives are reflected in the criteria they primarily score. For domains where both clinical and non-clinical perspectives are relevant (e.g., innovation, qualification), a joint review is conducted, and the final score is the average of both assessments.

## **4.1 Clinical Experts – Primary Responsibility**

| Domain | Sub‑criteria (examples) | Rationale |
| --- | --- | --- |
| Safety | Intraoperative, short‑term, long‑term adverse events | Direct procedural experience |
| Efficacy | Blood pressure reduction (office, ambulatory, night‑time) | Interpretation of clinical trial data |
| Patient Value | QoL improvement, medication adherence | Patient interaction and follow‑up |
| Qualification & Suitability | Technological maturity, ease of use, learning curve, technical support | Hands‑on device handling |
| Innovation | Adaptability to complex anatomy | Anatomical and procedural insight |

## **4.2 Non‑Clinical Experts – Primary Responsibility**

| Domain | Sub‑criteria (examples) | Rationale |
| --- | --- | --- |
| Cost | Direct medical costs, hospital perspective costs, societal costs | Budgeting and reimbursement knowledge |
| Management Value | Accessibility (supply chain), quality stability, brand reputation, reimbursement policy alignment | Procurement, logistics, and policy compliance |
| Qualification & Suitability | NMPA regulatory approval, ethical compliance | Regulatory and legal expertise |
| Innovation | Breakthrough device designation (domestic/international) | Understanding of regulatory innovation pathways |

## **5. Scoring Procedure**

### **5.1 Data Collection**

For each RDN product, gather evidence from: Published clinical trials and registries; NMPA approval documentation; Hospital procurement records (costs); Manufacturer technical specifications; Local reimbursement policy documents

### **5.2 Independent Scoring**

Each assessor independently scores the sub‑criteria assigned to Scoring standards, using the 0-10 scale. For products with no clinical evidence or insufficient evidence reporting, a neutral score of 5 should be assigned.

### **5.3 Consensus Meeting**

For sub‑criteria with score disagreement >5 points between assessors, a consensus discussion is held. If no consensus, the average score is used.

### **5.4 Score Averaging**

For sub‑criteria assessed by multiple experts, calculate the arithmetic mean (rounded to one decimal).

## **6. Weighted Score Calculation**

The overall value score for each RDN product is calculated as:

Overall Score=Σ(Global Weight × Score) for all third-level sub-criteria

Example: NMPA approval global weight = 2.96%. If scored 5, contribution = 0.0296 × 5 = 0.148.

Final Score Range: Maximum possible = 10 (all sub‑criteria scored 10); Minimum possible = 0 (all scored 0)
